# Relative impact of multi-layered genomic data on gene expression phenotypes in serous ovarian tumors

**DOI:** 10.1186/1752-0509-7-S6-S9

**Published:** 2013-12-13

**Authors:** Kyung-Ah Sohn, Dokyoon Kim, Jaehyun Lim, Ju Han Kim

**Affiliations:** 1 Department of Information and Computer Engineering, Ajou University, Suwon, Korea; 2 Seoul National University Biomedical Informatics (SNUBI), Div. of Biomedical Informatics, Seoul National University College of Medicine, Seoul 110799, Korea; 3 Department of Biochemistry and Molecular Biology, Pennsylvania State University, University Park, Pennsylvania, USA; 4 Systems Biomedical Informatics Research Center, Seoul National University, Seoul 110799, Korea

## Abstract

**Background:**

The emerging multi-layers of genomic data have provided unprecedented opportunities for cancer research, especially for the association study between gene expressions and other types of genomic features. No previous approaches, however, provide an adequate statistical framework for or global analysis on the relative impact of different genomic feature layers to gene expression phenotypes.

**Methods:**

We propose an integrative statistical framework based on a sparse regression to model the impact of multi-layered genomic features on gene expression traits. The proposed approach can be regarded as an integrative expression Quantitative Traits Loci approach in which not only the genetic variations of SNPs or copy number variations but also other features in both genomic and epigenomic levels are used to explain the expression of genes. To highlight the validity of the proposed approach, the TCGA ovarian cancer dataset was analysed as a pilot task.

**Results:**

The analysis shows that our integrative approach has consistently superior power in predicting gene expression levels compared to that from each single data type-based analysis. Moreover, the proposed method has the advantage of producing a substantially reduced number of spurious associations. We provide an interesting characterization of genes in terms of its genomic association patterns. Important genomic features reported in previous ovarian cancer research are successfully identified as major hubs in the resulting association network between heterogeneous types of genomic features and genes.

**Conclusions:**

In this paper, we model the gene expression phenotypes with respect to multiple different types of genomic data in an integrative framework. Our analysis reveals the global view on the relative contribution of different genomic feature types to gene expression phenotypes in ovarian cancer.

## Introduction

Cancer is a complex disease mainly characterized by uncontrolled proliferation and cell growth. Genes regulating differentiation and cell growth must be altered for a normal cell to transform into a cancer cell [1]. Expression of oncogenes or tumor suppressor genes promotes the malignant phenotype of cancer cells or inhibits cell division, development, or survival of cancer cell, respectively [1]. In many respects, a general survey of gene expression phenotypes serves as a proxy for the nature and breadth of phenotypic variation in human cancer [2,3]. In addition, gene expression is strongly associated with other types of genomic data in genomic level or epigenomic level [4].

In order to identify the relation between gene expression and other types of genomic data, there have been many attempts for integrative analyses between them. The expression quantitative trait loci (eQTL) approach, which integrates large-scale genotype data and expression profiles, has been established and offers a new perspective in biomedicine [5-8]. This approach treats gene expression profiles as quantitative traits or intermediate phenotypes and searches for genomic variation which can explain the variance of the molecular traits [9,10]. In addition to SNP data as a genome level, many integrative analyses between copy number variation and gene expression have been reported to identify genes that are associated with gene dosage [11-14]. In terms of epigenetic regulation, DNA methylation or histone modification can serve to regulate gene expression in cancer [15-18]. Furthermore, as one of the important regulators of gene expression, miRNA expression can be integrated with gene expression to identify the selective degradation or selective inhibition of translation [19-21].

Despite these efforts, however, it only reveals a limited view on the genomic mechanisms underlying cancer with only a pair of genomic data at hand. Recently, the emerging multi-layers of genomic data have provided unprecedented opportunities to identify the global view of relations between multi-layers of genomic data. The Cancer Genome Atlas (TCGA) is a large-scale collaborative initiative to improve understanding of cancer using multi-layers of genomic data. The TCGA research network recently published many notable papers on several cancers concerning an interim analysis of DNA sequencing, copy number, DNA methylation, miRNA, and gene expression data [22-26]. The International Cancer Genome Consortium (ICGC) is another multidisciplinary collaborative effort to characterize a comprehensive description of genomic, transcriptomic and epigenomic abnormalities in 50 different cancer types [27]. While the TCGA and ICGC open many opportunities to deepen the knowledge of the molecular basis of cancer [27-29], it is particularly important to access different levels of genomic data at hand for providing an enhanced global view on interplays between them.

**Figure 1:**
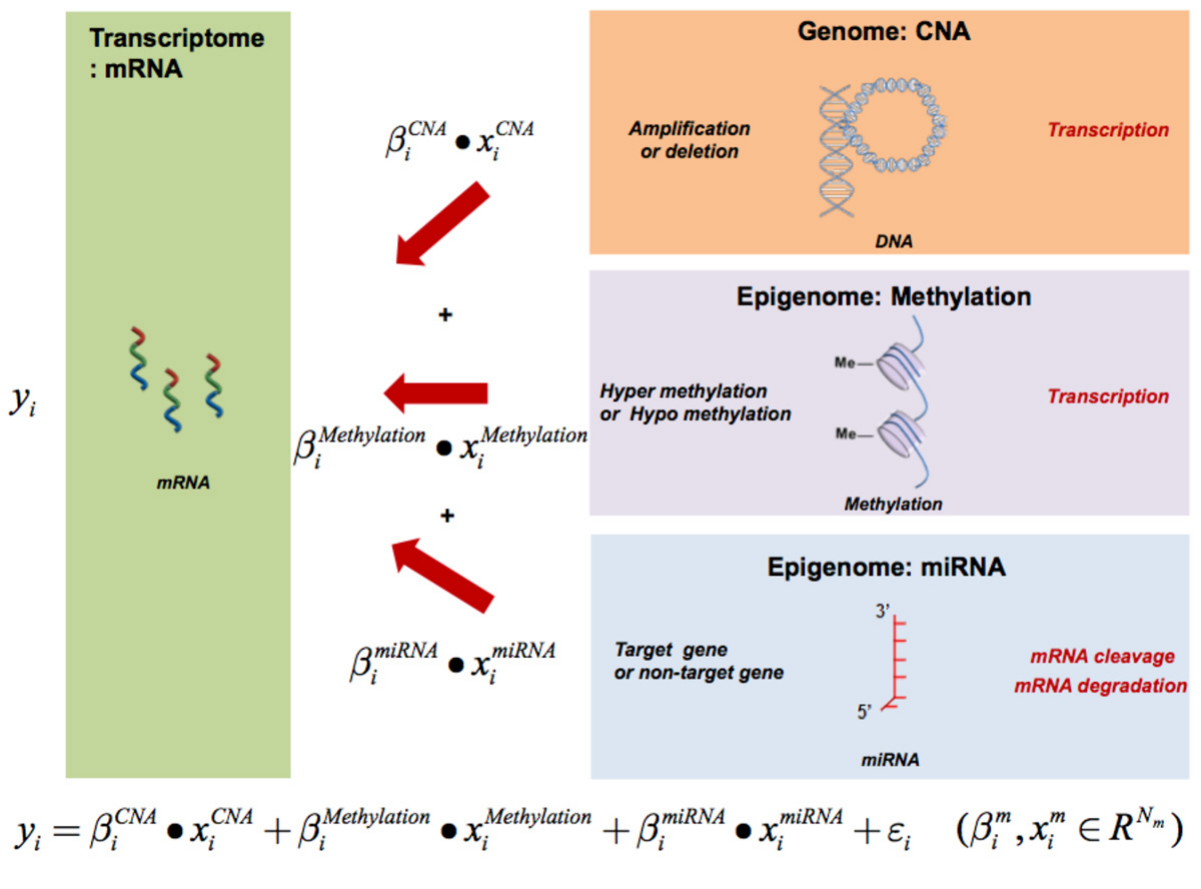
**The schematic overview of the integrative framework**. Different levels of genomic data such as copy number, methylation, or miRNA might affect gene regulation through either specific or synergistic fashion.

The emerging large-scale multi-layers of genomic dataset demand novel computational methods. There have been several integrative approaches for multi-layers of genomic data. For example, Chari *et al*. used the integrative analysis approach with multi-dimensional genomics data, enabling the understanding of mechanisms that disturb regulatory/signalling cascades and downstream effects [30]. Another relevant method, CNAmet, is an R package for integrative analysis of high-throughput copy number, DNA methylation, and gene expression data to identify genes that are amplified, hypomethylated and upregulated, or deleted, hypermethylated and downregulated [31]. In addition, other types of integrative methodological framework have been recently proposed to identify multi-dimensional regulatory modules from different levels of genomic data [32] or to combine different levels of genomic data for cancer clinical outcome prediction in the multiple-scale and the synergistic manner [33,34], which highlights the importance of integrative approaches utilizing multi-omics data systematically. However, to the best of our knowledge, there has not been any comprehensive analysis on the relative contribution of different genomic data to gene expression phenotypes, nor an adequate statistical approach to address this issue of elucidating gene expression phenotypes with more than two types of genomic data at hand. As different levels of genomic data such as copy number, SNP, methylation, or miRNA, might affect gene regulation through either specific or synergistic fashion, an integrative framework that incorporates all these different genomic features as potential regulators of gene expression will lead us to an enhanced global view on interplays between them (Figure 1). Simple correlation-based association tests will typically result in a large number of associations redundantly appearing across different types of genomic features. This makes it difficult to accurately measure the relative impact of each genomic feature type to gene expression traits. In this paper, we propose a sparse regression based framework for elucidating expression phenotype using different layers of genomic data as covariates.

In order to demonstrate the validity of the synergistic impact of multiple genomic data on gene expression phenotypes, ovarian cancer data from TCGA was used as a pilot task. Ovarian cancer (OV) is one of the most common gynecological malignancies, and is the 5^th ^leading cause of cancer mortality in women in the United States [35]. Understanding the molecular pathogenesis and underlying biology in ovarian cancer through the global view on interplays between different levels of genomic data is expected to provide guidance for improved prognostic indicators and effective therapies.

Through this pilot task, we validate that the predictive power of the proposed integrative model is consistently superior compared to those of the individual approaches that employ each type of genomic data separately. Moreover, the proposed framework is substantially more effective in reducing spurious associations between gene expression phenotypes and other genomic features. Comparison of the resulting association networks from these two approaches also supports the validity of the proposed framework. Based on this validation, we provide more focused analyses on the inferred association network to highlight the biological significance of our findings.

## Materials and methods

### Data

Datasets in ovarian cancer were retrieved from the Cancer Genome Atlas (TCGA) data portal (http://tcga-data.nci.nih.gov) (Table 1). The beta-value of Infinium methylation 27 BeadChip, ranged from 0 to 1, was used for DNA methylation data. After obtaining beta-values of methylation probes, the final matrix of DNA methylation was constructed by segmenting the 27,578 methylation probes into 9,219 gene features after removing zero-values across all the 381 samples. Level 3 data of gene expression and miRNA expression from TCGA were used as an expression signal of a gene or miRNA, containing 12,042 genes and 799 miRNAs, respectively. Copy number alteration (CNA) data were obtained from cBio Cancer Genomics Portal in order to use the results of GISTIC which attempts to identify significantly altered regions of amplification or deletion across sets of patients [36]. CNA data contains 54 significant cytoband regions with categorical values, -2, -1, 0, 1, or 2. As we use methylation, CNA, and miRNA data as covariates in our predictive statistical framework, we denote these three types of data as different *genomic feature types*.

**Table 1:** Data description

Cancer type	Data type	Platform	# Features (*d*) after preprocessing
OV	CNA	Agilent SurePrint G3 Human CGH Microarray Kit 1x1M	54
	Methylation	Infinium *humanmethylation27 *BeadChip	6,913
	miRNA	Agilent Human miRNA Microarray Rel12.0	799
	Gene expression	Affymetrix HT Human Genome U133 Array Plate Set	12,042

### Data preprocessing

To minimize the effect of heterogeneity in feature-wise distributions, type-specific preprocessing schemes are applied to each genomic feature set. Methylation data having the greatest number of features are non-specifically filtered by variance such that methylation features with lower 25% variance are removed from the feature set. We use all the 799 microRNA features without further filtering not only because the number of miRNA features is relatively small in comparison with that from methylation data, but also because the overall variance of microRNA expression data were relatively high. Copy number alteration data have unique characteristics in that the copy number alteration event typically occurs across long range of loci on a chromosome rather than on each single locus or short regions. After exploring several different feature representation alternatives, for example, those based on either probes or genes, we chose to use cytoband-based copy number alteration features, the output of GISTIC, showing the best performance through our analyses below.

We examine the impact of these resulting genomic features on each of the 12,042 gene expression traits. The feature values of all genomic data are finally normalized to have a zero mean and standard deviation of one across samples so that the relative impact of different genomic features on expression traits can be properly represented. Under this setting, let **y*_k _***denote an *N*-dimensional vector for expression traits of gene *k *in *N *= 381 individuals, and let **X*^{m} ^***denote an *N × J*^{*m*} ^feature matrix for *N *samples and *J*^{*m*} ^number of genomic features where *J*^{*m*} ^= 54, 6913, and 799 for *m*∈{*CNA, methylation, miRNA*}, respectively.

### Simultaneous feature selection and regression by a sparse regression

We first describe the baseline framework for modeling the effect from a single type of genomics features to gene expression traits. Typically, the association between genomic features and gene expression traits has been analyzed by a simple correlation test, either under a parametric assumption or in non-parametric way. As this relies on a pair-wise test between each single feature and each single gene, it is not capable of modeling the synergistic effect from multiple features to an expression trait. Moreover, the simple correlation measure tends to produce a large number of indirect genomic associations and does not reflect the possible interplays between potential regulators. The multiple testing issues caused by the huge number of pairwise tests also discourage the use of such a test for this type of integrative analysis.

Instead of this, we employ a sparse regression framework that has recently emerged as a powerful tool for detecting associations in a high-dimensional space. Under this model, the impact of *J *possible features *x*_1*i*_, ... , *x_Ji _*to a trait value *y_i _*is modeled as a multivariate linear regression as follows:

$$y_{i}=\beta_{0}+\beta_{1}\;x_{1i}+\beta_{2}\;x_{2i}+\ldots \beta_{J}\;x_{Ji}+\varepsilon_{i},\;\;\varepsilon_{i}\sim N\left(0,\sigma^{2}\right)$$

where *i *denotes the index for different samples. The *L*_1_-penalized regression framework called lasso [37] solves the following optimization problem to detect a relatively small number of effective covariates affecting the trait:

$$\text{min}\;{\text{Σ}}_{i}{\left(y_{i}-\left(\beta_{0}+\beta_{1}x_{1i}+\beta_{2}x_{2i}+\ldots \beta_{J}x_{Ji}\right)\right)}^{2}+\lambda \;{\text{Σ}}_{j}\text{|}\beta_{j}\text{|}$$

The second term of *L*_1_-penalty on **β **= (*β_1_, ..., β_J_*) induces a sparse solution by reducing the number of non-zero coefficients in **β**. The regularization parameter λ controlling the degree of sparsity is determined by cross-validation. Therefore, the solution given by lasso generates a set of a few features in association with the trait and the association strength of each effective feature *j *from *β_j_*. We adopt a Screen and Clean procedure [38] on top of lasso as our baseline statistical framework to allow further filtering of detected features based on *p*-values. We set the threshold for the *p*-values as 0.05 throughout our analysis.

We extend this baseline to an integrative model that deals with *M *different types of data as covariates assuming the following formulation:

$$y_{i}={\text{β}}^{\left\{1\right\}}•{x^{\left\{1\right\}}}_{i}+{\text{β}}^{\left\{2\right\}}•{x^{\left\{2\right\}}}_{i}+\ldots +{\text{β}}^{\left\{M\right\}}•{x^{\left\{M\right\}}}_{i}+\varepsilon_{i},\;\;\;\varepsilon_{i}\sim N\left(0,\;\;\sigma^{2}\right)$$

where **β**^{m}^, **x^{m}^**i ∈ R^J{m} ^for *m *= 1, ..., *M*.

Note that we excluded the intercept *β_0 _*because we already centered the data matrix to have zero mean column-wise. Through this formulation, a trait may be impacted by either one type of genomic features, or by multiple types of genomic features synergistically. Since the selected TCGA dataset provides three different types of genomic data as genomic features, the final optimization problem we solve is:

$$\begin{array}{c} \text{min}{\text{Σ}}_{i}{\left(y_{i}-\left({\text{β}}^{CNA}•{x^{CNA}}_{i}+{\text{β}}^{methlyation}•{x^{methlyation}}_{i}+{\text{β}}^{miRNA}•{x^{miRNA}}_{i}\right)\right)}^{2}+\\ \lambda \;\text{Σ}\left(\text{||}{\text{β}}^{CNA}\text{|}{\text{|}}_{\text{1}}+\text{||}{\text{β}}^{methlyation}|{|}_{1}+||{\text{β}}^{miRNA}|{|}_{1}\right) \end{array}$$

We denote the sparse solution of the above integrative setting by **β^{m}^**_integrative_.

As a base case, the aforementioned lasso-based Screen and Clean procedure is applied to each pair of (***X*^{*m*}^**,**y***_k_*) separately for *m*∈{*CNA, methylation, miRNA*}, and for each gene *k *= 1,...,12042. We denote the resulting coefficient matrix by **β^{m}^**_single_.

We validate the proposed integrative framework by comparing these two association networks implied by **β^{m}^**_integrative _and **β^{m}^**_single_. Note that both the 'integrative' approach and the 'single type'-based approach generate pairs of genomic associations between a genomic feature and a gene expression phenotype, and the strength of the association given by the magnitude of the corresponding regression coefficient.

## Results

### Predictive power of the integrative feature is consistently superior compared to that of each single type of genomic features

**Figure 2:**
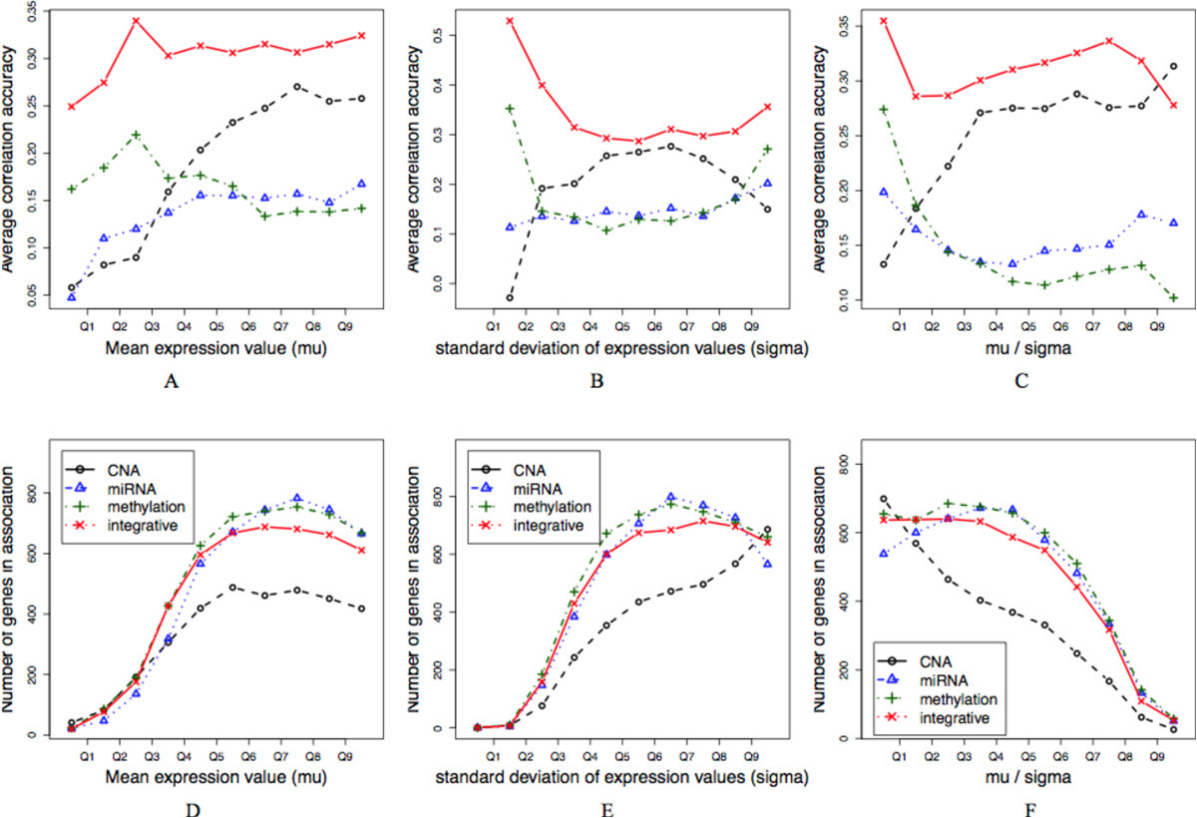
**Comparison of overall predictive power**. The average prediction accuracy of the proposed integrative method is compared with those using each genomic feature type separately (A,B,C). Genes are first partitioned into 10 equal-sized bins according to their average expression levels, standard deviation, and the ratio of the two, respectively, and then the result is displayed as a function of these deciles. The number of genes in each bin having at least one genomic association is also displayed in D,E,F.

One of the advantages of the sparse regression framework we adopt is that it is a predictive model and thus allows a quantitative performance evaluation. As a validation for the proposed integrative approach, we first compare the overall prediction accuracy of the integrative approach with those from each single genomic type based approaches using CNA, methylation, miRNA data separately. The average correlation coefficient between the actual gene expression levels and the predicted ones across samples is used as an accuracy measure. To examine the trend in the overall predictive power of each feature type, the genes are first partitioned into 10 equal-sized bins according to (a) the average expression levels µ across samples, (b) the standard deviation of the expression levels σ, and (c) the ratio of the two µ/ σ. Then we removed the predicted associations with association strength smaller than a threshold ρ of 0.1 to filter out less confident associations in both approaches. The prediction accuracies on the resulting genes are displayed in Figure 2 (A,B,C). The number of genes predicted to be in association with at least one feature is also displayed along the same deciles (Figure 2 D,E,F).

A number of interesting association patterns are clearly seen from Figure 2. First of all, the overall predictive power of the integrative feature is consistently superior to that from each single type of genomic features across the deciles. We also find that highly expressed genes (with higher µ) tend to be explained better by copy number alteration data, both before and after normalization by standard deviation (Figure 2 A,C). In contrast, variably expressed genes (with higher σ in Figure 2B, or more apparently, with smaller µ/σ in Figure 2C) tend to be explained better by the methylation features. This observation confirms the expectation that methylation plays a more dynamic role in regulating the gene expression through a dynamic epigenetic mechanism.

The number of genes having at least one genomic association is slightly smaller in case of the integrative framework than in a single type based approach using either methylation or miRNA data only. Considering the superior prediction accuracy of the integrative approach, we conjecture that this is because the indirect or spurious associations are effectively reduced in the integrative approach.

### Integrative approach is effective in reducing spurious associations

We next compare the number of genes specifically associated with each genomic data type and that of genes synergistically affected by multiple types of features. Recall that in our integrative approach, the expression level of a gene *k *is represented as a linear combination of CNA, miRNA, and methylation features using three coefficient vectors ***β**^CNA^, **β**^miRNA^*, and ***β**^methylation^*. If at least one element in the estimated ***β**^m ^*is non-zero, we can say the gene is associated with the genomic feature type *m*. Therefore, a certain gene may be associated with more than one genomic feature type.

**Figure 3:**
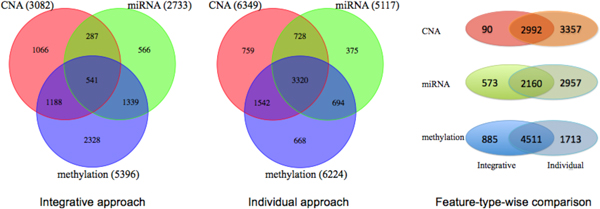
**Comparison of the number of genes specifically impacted by single type of features and synergistically affected by multiple types of data**. The number of genes impacted by each type of genomic data is presented as a Venn diagram. The integrative approach predicts a significantly smaller number of genes synergistically impacted by multiple types of data, and a greater number of genes that are specifically impacted by only one genomic data type, in comparison with the single data type based analysis. The integrative approach produces substantially less number of spurious associations in general, which can lead to more accurate characterization of genes based on the genomic association.

The summary for the number of genes impacted by each genomic feature type is presented in Figure 3. First, the number of genes having at least one genomic association with each feature type is smaller in the case of the integrative approach, for example, we find 3,082 CNA associated genes versus 6,349 such genes in the integrative and the single type based (of CNA-only) analyses, respectively. As already mentioned above regarding the predictive accuracy, this appears to be because of the fewer number of false positives produced in the integrative approach.

Furthermore, the single-type based approach estimated a very large number of genes that are impacted synergistically by all the three feature types, that is, 3,320 among total 12,042 genes, and more than half of either CNA, miRNA, or methylation associated genes. This seems to be a clear over-estimation of such genes. In contrast, the proposed integrative approach yields a substantially smaller number and fewer fractions of such synergistically impacted genes (541 genes). As a result, a greater number of genes fall into the class impacted specifically by only one genomic type under our integrative framework (e.g. 1,066 CNA-only-associated genes, versus 759 such genes in the single type-based analysis) even with a smaller number of total genes in association. This highlights the potential utility of our integrative approach for characterizations genes based on the genomic association and for the investigation of relative contribution of different genomic feature types as well.

### Heterogeneous genomic association network from the integrative approach has better modularity

**Figure 4:**
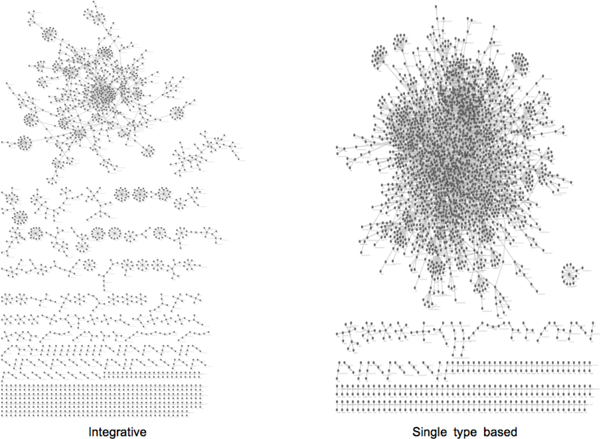
**Comparison of the heterogeneous association networks recovered from the integrative and individual approaches**. The association networks having heterogeneous types of genomic features and expression traits as nodes and their associations as edges are drawn using Cytoscape. Overall, the one from the proposed approach reveals enhanced network modularity.

We provide a global outlook of the association networks estimated from both the integrative approach and the single type based approach. Figure 4 shows the heterogeneous genomic association networks in which features from copy number alteration, methylation, miRNA or gene expression data are represented as nodes and the edges are constructed from the estimated non-zero regression coefficients **β^{m}^**_integrative _and **β^{m}^**_single_. For better visualization, the network edges were further filtered with a threshold ρ = 0.3 and nodes without any connected edge were removed. The resulting networks reveal very different global topologies such as the number of connected components or the clustering coefficients. Overall, the one from the proposed approach clearly has better modularity as illustrated in Figure 4, which may imply more functionally coherent network modules in it.

We performed functional enrichment test with respect to GO Biological Process for the set of genes in the largest connected component in each network. The enriched GO BP terms with the smallest FDR corrected *p*-values are largely related to defense response and immune system in both approaches. For example, the most significant term was GO:6952 *defense response*, and GO:9611 *response to wounding*, with FDR corrected *p*-values of 2.65e-23 and 1.13e-20, respectively, in the integrative and the single-type based approach. The integrative approach also detected GO:42330 *taxis *and GO:6935 *chemotaxis *(FDR corrected *p*-values of 9.35e-12 and 9.35e-12, respectively) as the 9^th ^and 10^th ^most significant terms, which has known to be essential in cancer progression and metastasis. In contrast, the single type-based analysis tends to produce more broad terms such as GO:48856 *anatomical structure development *or GO:48731 *system development *(4^th ^and 5^th^, FDR corrected *p*-values of 3.53e-18 and 1.72e-17, respectively) other than the aforementioned common terms.

### Relative contribution of each genomic feature type to gene expression phenotypes

**Figure 5:**
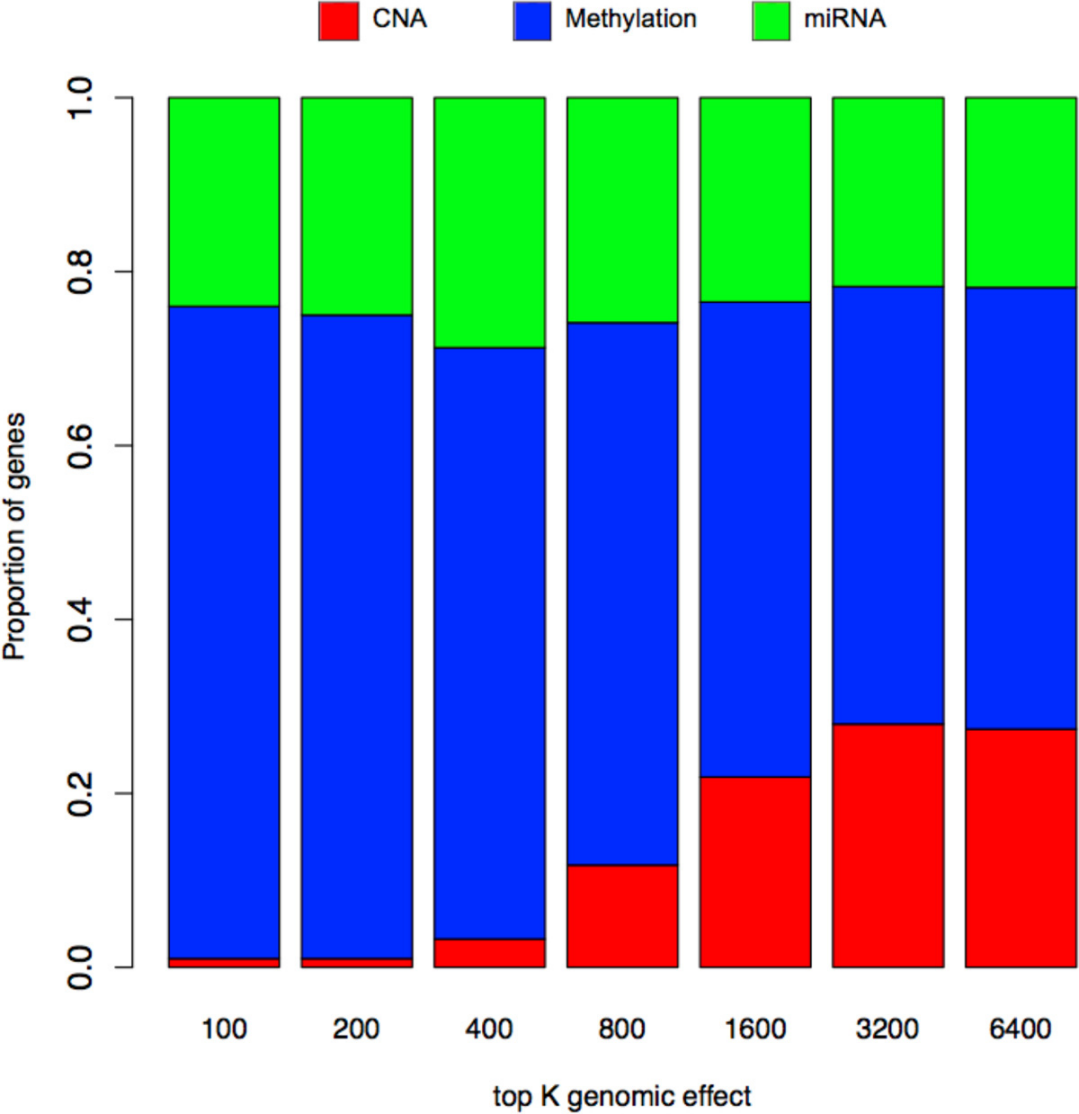
**Relative contribution of each genomic dimension to gene expression traits**. We investigate the proportion of genes in association with each genomic type in the top K associations having the greatest association strength. Methylation appears to have the dominant role in expression regulation, although miRNA and copy number alteration also has significant impact on gene expressions, especially considering the smaller number of used features.

We now perform more focused analysis on the association network estimated under the proposed framework. In Figure 5, the relative contribution of each genomic feature type to gene expression traits is characterized by looking at an increasing number of detected genomic associations. Specifically, the proportion of genes in association with each genomic feature type calculated using the top *K *strongest genomic effects are shown for *K *= 100,200,400,800,1600,3200, and 6400.

First, the effect of methylation feature was the most dominant overall. Among the top 100 genomic associations, only one association was occurring with copy number alteration feature (CCNE1 expression and copy number alteration at 19q12), 24 were with miRNAs, and the remaining 75 were with methylation features. We noticed that among 75 methylation-mediated associations, 29 were the trivial associations of a gene with the methylation near the same gene. This may have caused to overestimate the proportion of methylation-affected genes when we consider too small number of top signals. As we increase the number of top genomic effects *K*, the proportion of CNA-associated genes steadily increases from 0.01 to 0.27 while that of methylation-associated genes decreases from 0.75 to 0.5 until *K *= 3200 and remains similar after that. The proportion of miRNA-associated genes remains similar across *K*, ranging from 0.22 to 0.28. While methylation seems to contribute the most to gene expression variation in ovarian cancer, the impacts of CNA and miRNA are also surprisingly significant considering the substantially smaller number of used features (54 and 799, respectively) than that of methylation features (6913).

### Hub genomic features and the functional characterization of the co-affected genes

We further zoom into the association network and examine the top 10 hub genomic features impacting the largest number of genes. The hub genomic features and the most significant GO terms and SP-PIR (Swiss-Prot and Protein Information Resource) keywords for the set of associated genes are listed in Table 2. For instance, miRNA-768-5p, which has previously shown to play an important role in ovarian cancer progression [39], has the greatest number of 303 genes as neighbours. miRNA-768-5p was also identified as microRNA signatures of tumor-derived exosomes for the diagnostic biomarkers of ovarian cancer [40]. The functional enrichment test using DAVID shows that the affected genes of miRNA-768-5p are the most significantly enriched with SP-PIR keyword *acetylation *(FDR corrected *p*-value = 1.4e-19). In addition, miRNA-29-a is well known of oncosuppressor miRNA, which is frequently lost or down-regulated in cancer so that target oncoproteins like CDK6, MCL1, or BCL-2 can be upregulated [41]. Among the top 10 hub features, 7 of them were copy number alteration features, supporting the significance of copy number alteration event in cancer progression and treatment. CNA features affect several genes associated with acetylation, phosphoprotein, or nucleus. Methylation of Sprouty-4 (SPRY4), an inhibitor of the receptor-transduced mitogen-activated protein kinase (MAPK) signalling pathway, has been detected in prostate cancer [42]. Methylation at SPRY4 affects 119 genes in downstream, which also is related to *acetylation*.

**Table 2:** Top 10 hub genomic features impacting the largest number of genes.

Genomic feature	Type	*N*	Most significant GO term	*p*-value (FDR)	Most significant SP-PIR keyword	*p*-value (FDR)
hsa.miR.768.5p	miRNA	303	GO:0005739 mitochondrion	8.67E-09 (1.18E-05)	acetylation	1.10E-22 (1.40E-19)
hsa.miR.29a	miRNA	154	GO:0007049 cell cycle	7.99E-20 (1.21E-16)	cell cycle	7.30E-17 (1.44E-13)
SPRY4	methylation	119	GO:0003723 RNA binding	3.19E-4 (0.4197)	acetylation	1.09E-6 (0.0013)
16p13.3	CNA	117	GO:0031974 membrane-enclosed lumen	4.10E-5 (0.0506)	iron-sulfur	9.98E-5 (0.1257)
12q23.1	CNA	113	GO:0070013 intracellular organelle lumen	4.29E-5 (0.0534)	acetylation	2.62E-6 (0.0032)
1q42.3	CNA	113	GO:0012505 endomembrane system	7.3E-4 (0.8908)	phosphoprotein	4.71E-6 (0.0059)
6p21.1	CNA	109	GO:0042974 retinoic acid receptor binding	4.86E-8 (6.38E-5)	nucleus	1.84E-5 (0.0228)
17q25.3	CNA	108	non-membrane-bounded organelle	0.0092 (11.23)	acetylation	6.39E-9 (8.03E-6)
1p36.11	CNA	103	GO:0016071 mRNA metabolic process	0.0046 (6.86)	phosphoprotein	1.31E-4 (0.1632)
19p13.12	CNA	102	GO:0043232 intracellular non-membrane-bounded organelle	1.24E-5 (0.0150)	nucleus	1.24E-5 (0.0149)

The most significantly enriched GO terms and SP-PIR keywords for the set of associated genes are listed together.

## Discussion and conclusion

We proposed to elucidate the gene expression phenotypes with multiple different types of genomic features together to gain better insight on the global genomic mechanism underlying cancers. Through the analysis of TCGA ovarian cancer dataset, we validated the proposed integrative framework in various aspects. The proposed approach provided a systematic view on the relative contribution of different types of genomic data on the expression of genes. Since different levels of genomic data might affect gene regulation through either partly independent or partly complementary fashion, proposed framework that incorporates all these different genomic features as potential regulators of gene expression will lead us to an enhanced global view on interplays between them. Understanding the molecular pathogenesis and underlying complex mechanisms in ovarian cancer through the global view on interplays between them is expected to provide guidance for improved prognostic indicators and effective therapies [33].

The proposed approach may be regarded as an *integrative eQTL *approach in which not only the genetic variations of SNPs but also other features in both genomic and epigenomic levels are used to explain the expression of genes. The original purpose of eQTL is to search genomic variations which can explain the variance of the gene expression as an intermediate phenotype. Thus, it can be conceptually extended to integrative approach with other levels of genomic features in order to better explain gene expression as a phenotype level. Since TCGA does not provide SNP data publicly, we excluded SNPs from our analysis. Thus, we used copy number data as a feature in genome level in this study. However, integration with SNP data will provide opportunities to investigate the genetic associations as well as the epigenetic associations in a principled way.

One limitation of the proposed approach is the parametric assumption of normal distribution for genomic features, which is not valid in general. We leave this investigation about the deviation from the parametric assumption and possible improvement as our future work. Another interesting direction for further research would be the integration with existing biological knowledge. Systematic schemes for the choice, representation, and incorporation of such knowledgebase remains as our further research plan.

We used ovarian cancer dataset, which is one of the datasets in the first phase of TCGA project, as a pilot task for the study. However, TCGA has been generating additional cancer genomic data for about 25 tumor types as the second phase of the project, mainly sequencing-based data. Since our proposed method is flexible to use any kind of multi-omics data, it will be easily extended to other cancer types as a future work.

## Competing interests

All authors declared that there is no conflict of interest in this research.

## Authors' contributions

KS and DK designed and developed the study and wrote the manuscript. KS, DK, and JL produced the experimental results and interpreted the results. JHK provided intellectual guidance and mentorship and wrote the manuscript. All authors read and approved the final manuscript.

## Declarations

The publication costs for this article were funded by the National Research Foundation of Korea (NRF) within Basic Science Program (grant no. 2012-0000994).

This article has been published as part of *BMC Systems Biology *Volume 7 Supplement 6, 2013: Selected articles from the 24th International Conference on Genome Informatics (GIW2013). The full contents of the supplement are available online at http://www.biomedcentral.com/bmcsystbiol/supplements/7/S6.
